# Primary care consultation patterns before suicide: a nationally representative case–control study

**DOI:** 10.3399/BJGP.2023.0509

**Published:** 2024-05-28

**Authors:** Danah Alothman, Sarah Lewis, Andrew W Fogarty, Timothy Card, Edward Tyrrell

**Affiliations:** School of Medicine, University of Nottingham, Nottingham.; School of Medicine, University of Nottingham, Nottingham.; School of Medicine, University of Nottingham, Nottingham.; School of Medicine, University of Nottingham, Nottingham.; School of Medicine, University of Nottingham, Nottingham.

**Keywords:** consultation, family practice, general practice, primary health care, suicide

## Abstract

**Background:**

Consultation with primary healthcare professionals may provide an opportunity to identify patients at higher suicide risk.

**Aim:**

To explore primary care consultation patterns in the 5 years before suicide to identify suicide high-risk groups and common reasons for consulting.

**Design and setting:**

This was a case–control study using electronic health records from England, 2001 to 2019.

**Method:**

An analysis was undertaken of 14 515 patients aged ≥15 years who died by suicide and up to 40 matched live controls per person who died by suicide (*n* = 580 159), (*N* = 594 674).

**Results:**

Frequent consultations (>1 per month in the final year) were associated with increased suicide risk (age- and sex -adjusted odds ratio [OR] 5.88, 95% confidence interval [CI] = 5.47 to 6.32). The associated rise in suicide risk was seen across all sociodemographic groups as well as in those with and without psychiatric comorbidities. However, specific groups were more influenced by the effect of high-frequency consultation (>1 per month in the final year) demonstrating higher suicide risk compared with their counterparts who consulted once: females (adjusted OR 9.50, 95% CI = 7.82 to 11.54), patients aged 15–<45 years (adjusted OR 8.08, 95% CI = 7.29 to 8.96), patients experiencing less socioeconomic deprivation (adjusted OR 6.56, 95% CI = 5.77 to 7.46), and those with psychiatric conditions (adjusted OR 4.57, 95% CI = 4.12 to 5.06). Medication review, depression, and pain were the most common reasons for which patients who died by suicide consulted in the year before death.

**Conclusion:**

Escalating or more than monthly consultations are associated with increased suicide risk regardless of patients’ sociodemographic characteristics and regardless of the presence (or absence) of known psychiatric illnesses.

## Introduction

Primary care plays a vital role in suicide prevention as it is usually the first point of healthcare contact and is the most frequently used healthcare service by patients who died by suicide before their death.^1^ Previous international studies have shown an increased likelihood of GP consultation in people who died by suicide, compared with the general population.^2^^–^^4^

In the UK, risk of suicide has been shown to rise with increasing primary care consultation frequency in the year before suicide,^5^^–^^8^ but very little is known about whether this pattern extends beyond the final year before suicide. Sociodemographic characteristics^1^^,^^9^ as well as psychiatric diagnoses^10^^,^^11^ have been shown to influence rates of consultation with primary care. However, how suicide risks in relation to primary care consultation may differ across various sociodemographic subgroups or in those with (or without) psychiatric diagnoses, to the authors’ knowledge, have not yet been studied.

Mughal and colleagues used inquest records, confidential enquiry, serious incident, and criminal justice system reports to examine predefined reasons for which middle-aged males consulted primary care in the 3 months before suicide.^12^ These patients were more likely to have consulted for self-harm, mental health problems, and job-related issues. To the authors’ knowledge, primary care data have not previously been used to explore common reasons for primary care consulting in the year before suicide, and this provides an opportunity to do so in the wider population in a more explorative manner.

In the current study, therefore, a large population-based sample in England was used to analyse patterns of consultation with primary care professionals to identify patients at higher risk of suicide. Specifically the aim was to:

- quantify the risk of suicide in relation to the number of primary care consultations in the year before suicide, including how any association between suicide and consultation frequency differs across important sociodemographic and clinical subgroups;
- describe patterns of consultation in the 5 years before suicide; and
- explore common reasons why patients who died by suicide consulted in the final year.

**Table:** How this fits in

Although increased primary care utilisation in the preceding year has been linked with death by suicide, longer-term consulting patterns and primary care-recorded reasons for consulting have not been previously examined. This large, nationally representative sample from England showed rates of consulting among patients who died by suicide continuously rose in the 5 years before suicide, especially in the last 3 months. Suicide risk was significantly increased among those who consulted more than once every month in the final year, irrespective of any sociodemographic characteristics and irrespective of the presence (or absence) of known psychiatric comorbidities. Common reasons why patients who died by suicide consulted before their death included medication review, depression, and pain.

## Method

### Data source

The current study used data from Clinical Practice Research Datalink (CPRD) GOLD and CPRD Aurum, large longitudinal datasets of primary care healthcare records, with a broad representation of the UK population in terms of age, sex, and ethnicity.^13^^,^^14^ The current analysis was restricted to a subset of 75% (*N* = 70 065 533) of English CPRD practices with linkage to national databases on deaths and socioeconomic deprivation.^13^ Records of suicide were obtained from the Office for National Statistics (ONS), the gold standard for obtaining information related to death by suicide in England.^15^ Deprivation measures based on patients’ home postcode were derived from the Index of Multiple Deprivation (IMD) — a composite score encompassing seven domains to assess socioeconomic deprivation.^16^ Records of psychiatric illnesses were derived from both CPRD and the Hospital Episode Statistics (HES) database.^17^

### Study design and population

A case–control study was conducted using data from England from between 1 January 2001 and 31 December 2019. Patients with records in CPRD were eligible for inclusion if: their records were deemed to be of ‘acceptable’ quality (and for CPRD GOLD only if their primary care practice was deemed ‘up-to-standard’); they were eligible for linkage with the other databases outlined above; they were aged ≥15 years; they had a minimum of 1 year of complete records in CPRD before suicide. All suicide deaths among eligible patients occurring during the study period were included. There were 5 years of consultation data before this were extracted for the case patients (those who died by suicide) and their matched control patients.

#### Case group

All eligible patients with an ONS record of suicide (or undetermined death) during the study period, identified from the following International Classification of Diseases 10th revision (ICD-10) codes: X60–84, Y10 – 34 (excluding Y33.9), Y87.0, and Y87.2 were included. Undetermined deaths have been reported to be mostly suicide deaths and their inclusion is recommended in studies of suicide to reduce false-negative misclassification of suicide deaths.^18^ Dates for patients who died by suicide refer to the date of registration of death in the ONS.

#### Control group

For every person who died by suicide, up to 40 live control patients who were registered at the same GP practice as the corresponding case patient were randomly selected from the eligible cohort. To allow longitudinal sampling, people in the control group were required to be alive and available in the dataset on the date of suicide of their corresponding case patient (risk-set sampling), henceforth known as the index date for the control patient. Assessments of exposure were made in relation to the index date for control patients just as they were in relation to the date of suicide for the case patient. The authors of the current study successfully identified 40 matched control patients for all but 275 suicide case patients (1.9% of the case group) for whom the authors were able to select between 9 and 39 matched control patients each.

### Exposure

#### Consultation

Information on the nature, location, and staff involved in clinical contacts as well as the clinical reasons recorded for contacts was abstracted from CPRD records. For this study only active encounters between patients and primary healthcare providers, including telephone consultations, were included. Consultations with any clinical member of the primary care team (including nurses, physiotherapists, and so on) were included. Letter, text or email encounters, or encounters outside primary care (that is, hospital or emergency department) were excluded.

#### Patterns of consultation

Information was collated on the frequency of consultations (for any reason) per year up to 5 years before the suicide/index date and per month in the final year before suicide (non-overlapping periods).

As the inclusion criteria of the study involved patients who had at least 1 year of complete records before the suicide/index date, for consultation patterns beyond the final year, the authors examined the frequency of consultations per annum in the subset of patients having complete records in the year under examination.

#### Reasons for consultation

Common reasons for consultations were explored using CPRD medical codes (in CPRD GOLD) and medical code IDs (in CPRD Aurum) by matching them to their corresponding medical terms (descriptions of conditions). These included any reason for consulting, not just those obviously linked to suicide. As medical concepts in CPRD have a complex and broad array of medical terms, a systematic iterative approach was followed to identify common reasons for consultation. This involved exploring frequently recorded medical terms, removing extraneous terms, and unifying medical terms that reflect analogous or related concepts. The lists for grouping of medical concepts/terms were reviewed and approved by all authors. The 10 most common reasons for consultation were delineated by:

- period prevalence of reason for consultation, that is, by the proportion of case patients and control patients with at least one record of a consultation for that reason in the final year; and
- ‘total’ recorded consultations for that reason in the final year.

### Covariates

Covariates in the current study were sex, age (at suicide/index date), IMD (available as deciles), and psychiatric illness. In total, 0.5% of patients (3042 of 594 674 patients) had missing IMD data, which were handled using multiple imputation by chain equations. Records of history of psychiatric illnesses were collected from both CPRD and HES (to improve ascertainment^19^). These included: affective disorders (including major depressive disorder); schizophrenia spectrum (and other psychosis); anxiety (including obsessive–compulsive disorders); personality disorders; eating disorders; sleep disorders; and substance misuse. These data were categorised into a binary indicator of the presence or absence of any psychiatric illness. Supplementary Information S1 and Supplementary Tables S1–S3 provide a full list of terms and codes used for identifying psychiatric illness.

### Analysis

Odds ratios (ORs) were estimated using a conditional logistic regression model. Given that the current study used risk-set sampling, ORs can be interpreted as rate ratios.^20^ As non-attendees and frequent attendees to primary care have previously been reported to be high-risk groups for suicide,^7^ the authors of the current study considered, therefore, that patients who consulted once were likely to be at lowest risk and were hence the reference group in the analysis. The frequency of consultations in the final year were fitted into four categories, guided by a likelihood ratio test (LRT) suggesting a non-linear association. The four categories, determined as a priori categories for analysis, were: no consultation in the final year; 1 consultation; 2–12 consultations; and ≥13 consultations.

Age (at suicide/index date) and sex were considered a priori confounders and age was fitted categorically into 10 equally sized decile groups (see Supplementary Information S2, for further information on age categorisation). Further adjustment for psychiatric illness (or other medical conditions) was avoided because the authors expect that those conditions primarily drive the association between consultations and suicide risk, and the aim was to use patterns of consultation (for any reason) as suicide markers to identify high-risk groups. Effect modification by sociodemographic characteristics and by psychiatric illness were tested using the LRT by fitting an interaction term.

A post hoc analysis was performed separating suicide risk in patients who consulted between two to six times (once or less than once every 2 months) and those who consulted between seven to 12 times (more than once every 2 months) in the final year. Further analysis was also performed exploring common reasons for primary care consulting in the 3 months as well as in the last month before the suicide or index date (Supplementary Information S3, Supplementary Table S4, and Supplementary Table S5). Stata version 17 was used for data analysis.

### Results

The dataset included 594 674 eligible patients: 14 515 (2.4% of the sample) who died by suicide (case group) and 580 159 (97.6%) who were control patients (control group). In the year leading up to suicide, 12 498 (86.1%) and 448 427 (77.3%) of the case group and the control group, respectively, had consulted at least once with primary care professionals. Among those who consulted in the final year, the median was 10 consultations (interquartile range [IQR] 4–21) for those dying by suicide and six consultations (IQR 3–13) for the control group (*P*<0.0001 based on the Wilcoxon rank sum test) (Table 1).

**Figure 1.**
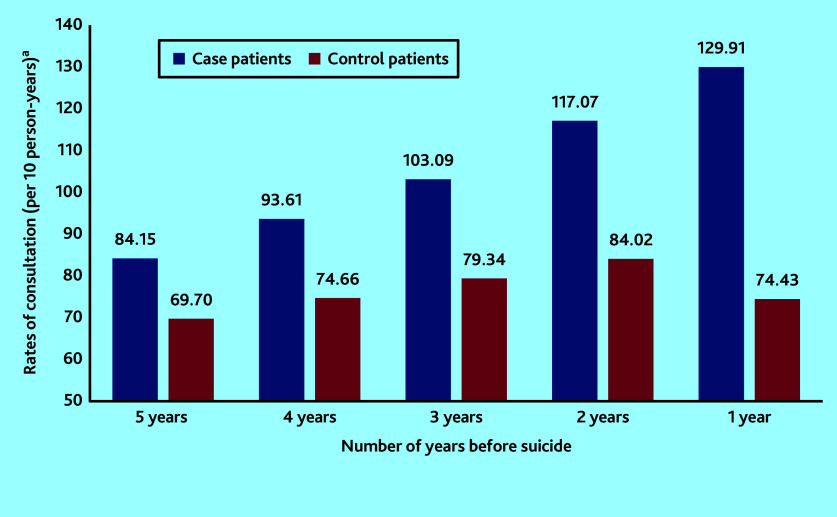
Comparison of rates of consultation with primary care professionals per annum among the case group and the control group in the 5 years before date of death by suicide. ^a^Frequencies of consultations and rates have been calculated in the subset of individuals with complete data in the year being examined.

**Figure 2.**
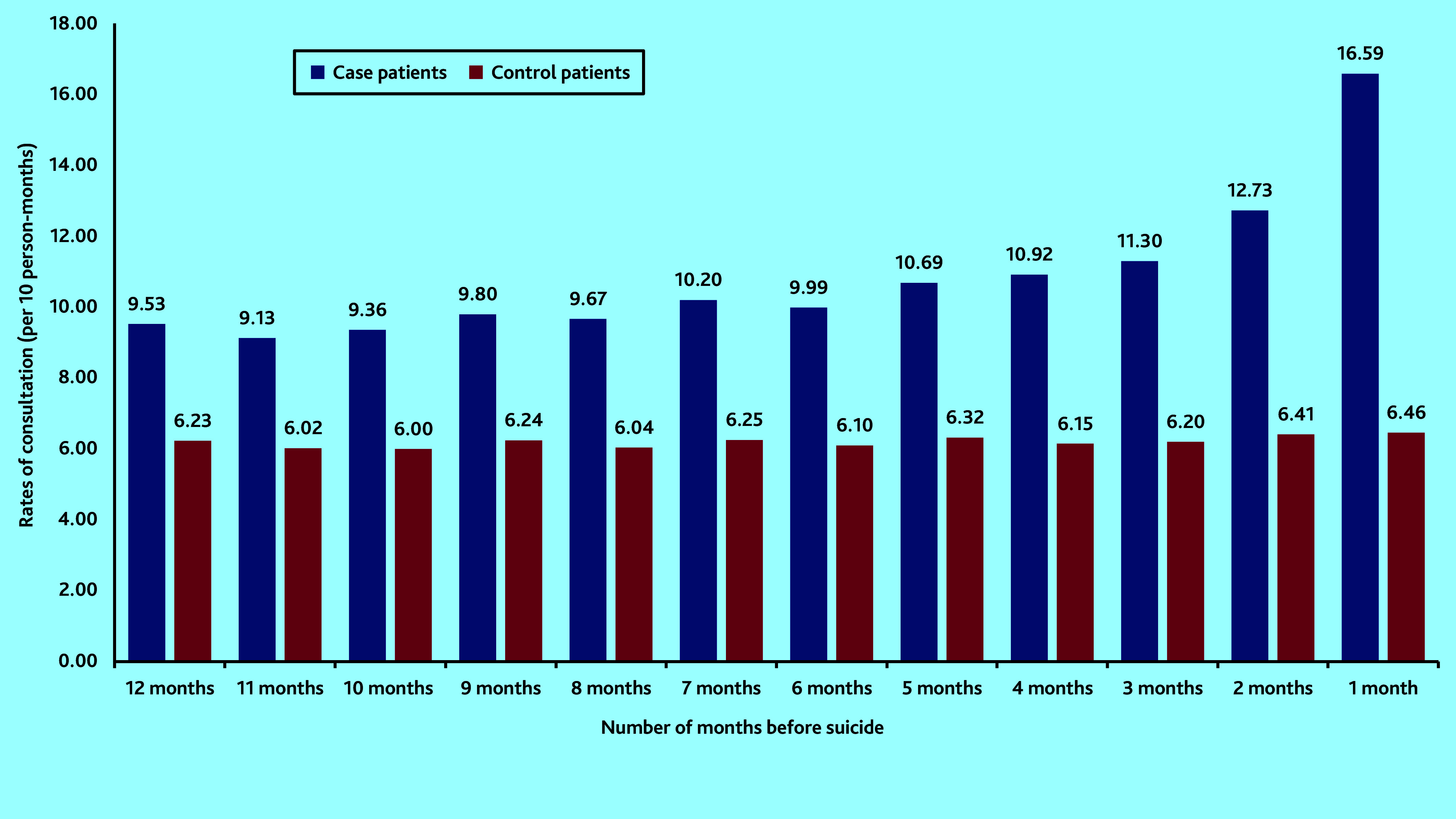
Comparison of rates of consultations with primary health care per month among the case group and the control group in the year leading up to the date of death by suicide.

Figure 1 shows that rates for those dying by suicide were on an increasing trend per year, in the 5 years before suicide. In the final year consultations particularly increased in the 3 months before suicide, with the highest consultation rate in the month before death (Figure 2). For the control group, rates of consultation were considerably more stable over time.

**Table 1.** Descriptive statistics for patterns of consultation in the year before suicide

**Pattern of consultation**	**Case group (*n* = 14 515)**	**Control group (*n* = 580 159)**
**Did not consult at all, *n* (%)**	2017 (13.9)	131 732 (22.7)
**Consulted at least once, *n* (%)**	12 498 (86.1)	448 427 (77.3)
**Total consultations, *n* (rate per 10 person-years)**	188 562 (129.9)	4 318 187 (74.4)
**Total consultations among those who consulted, median (IQR)^a^**	10 (4–21)	6 (3–13)
**Time in days from last consultation to suicide/index date among those who consulted, median (IQR)^a^**	10 (0–47)	43 (13–116)

a P*<0.0001 for differences in median between the case group (who died by suicide) and the control group according to Wilcoxon rank sum test. IQR = interquartile range.*

As demonstrated in Table 2, patients who did not consult with primary care professionals in the year leading up to suicide were at a significantly lower risk of suicide than those who consulted once (adjusted OR 0.65, 95% confidence interval [CI] = 0.60 to 0.72). Conversely, in the year before suicide, patients who consulted ≥13 times (more than once per month) were 5.88 times (95% CI = 5.47 to 6.32) more likely to die by suicide than those who consulted once.

A post hoc analysis showed that patients who consulted two to six times in the final year were at 1.47 times (95% CI = 1.37 to 1.58) higher risk of suicide and those who consulted seven to 12 times at 2.55 times (95% CI = 2.37 to 2.75) higher risk of suicide, than those who consulted once.

**Table 2.** Relative risk of suicide in patients who consulted with primary healthcare professionals in the year before suicide in comparison with those who consulted once in the final year

**Consultations in the year before suicide by demographic characteristic, *N*^a^**	**Adjusted OR (95% CI)**	***P*-value**
**All^b^**		<0.0001
Reference	1	
0	0.65 (0.60 to 0.72)	
2 to 12	1.77 (1.66 to 1.90)	
≥13	5.88 (5.47 to 6.32)	

**Sex^c^**		<0.0001
Males		
Reference	1	
0	0.62 (0.57 to 0.68)	
2–12	1.79 (1.67 to 1.93)	
≥13	5.30 (4.89 to 5.74)	
Females		
Reference	1	
0	0.95 (0.76 to 1.20)	
2–12	2.14 (1.77 to 2.60)	
≥13	9.50 (7.82 to 11.54)	

**Age^d^ (non-overlapping groups)**		<0.0001
15 to <45 years		
Reference	1	
0	0.66 (0.60 to 0.73)	
2–12	1.88 (1.72 to 2.06)	
≥13	8.08 (7.29 to 8.96)	
45 to <75 years		
Reference	1	
0	0.73 (0.64 to 0.83)	
2–12	1.50 (1.34 to 1.67)	
≥13	4.25 (3.80 to 4.76)	
≥75 years		
Reference	1	
0	0.41 (0.24 to 0.69)	
2–12	1.51 (1.00 to 2.26)	
≥13	2.86 (1.90 to 4.31)	

**Socioeconomic deprivation**		<0.0001
Least deprived (IMD 1–4)		
Reference	1	
0	0.63 (0.55 to 0.72)	
2–12	1.82 (1.62 to 2.06)	
≥13	6.56 (5.77 to 7.46)	
Moderate deprivation (IMD 5–7)		
Reference	1	
0	0.61 (0.53 to 0.71)	
2–12	1.65 (1.46 to 1.87)	
≥13	5.27 (4.61 to 6.02)	
Most deprived (IMD 8–10)		
Reference	1	
0	0.70 (0.61 to 0.80)	
2–12	1.80 (1.60 to 2.03)	
≥13	5.39 (4.75 to 6.12)	

**Psychiatric illnesses**		<0.0001
No known psychiatric illness		
Reference	1	
0	0.72 (0.65 to 0.81)	
2–12	1.07 (0.97 to 1.18)	
≥13	1.61 (1.42 to 1.84)	
Psychiatric illness		
Reference	1	
0	0.83 (0.74 to 0.94)	
2–12	1.73 (1.57 to 1.92)	
≥13	4.57 (4.12 to 5.06)	

a *The number of consultations in this table refer to the frequency of consultations in the year before suicide/index date. Multiple imputation was used to deal with missing data on IMD.*

b *Adjusted for sex and age at suicide/index date.*

c *Adjusted for age at suicide/index date.*

d *Adjusted for sex. IMD = Index of Multiple Deprivation. OR = odds ratio.*

Age, sex, socioeconomic deprivation (IMD), and psychiatric illness were all significant modifiers of effect (*P*-value for interaction *P*<0.0001) (Table 2). Although suicide risk in those who consulted frequently (≥13 times in the final year) was increased across all sociodemographic groups and in those with and without psychiatric illnesses, the effect of frequent consultations in increasing suicide risk was more prominent in: females consulting ≥13 times (adjusted OR 9.50, 95% CI = 7.82 to 11.54) compared with females who consulted once; younger patients aged 15 to <45 years consulting ≥13 times (adjusted OR 8.08, 95% CI = 7.29 to 8.96) compared with younger patients who consulted once; those experiencing least socioeconomic deprivation (IMD 1–4) consulting ≥13 times (adjusted OR 6.56, 95% CI = 5.77 to 7.46) compared with those experiencing socioeconomic deprivation who consulted once; and in those with known psychiatric conditions consulting ≥13 times (adjusted OR 4.57, 95% CI = 4.12 to 5.06) compared with those with known psychiatric conditions who consulted once.

Medication reviews and requests, depression, and symptoms of pain were the top three reasons (whether by period prevalence or total records) for which patients who died by suicide consulted in the year before death (Table 3). These remained unchanged when considering just the final 3 months before suicide (Supplementary Table S4). However, in the last month, patients who died by suicide mostly consulted for depression, medication review and requests, and self-inflicted injury (whether by period prevalence or total records) (Supplementary Table S5).

**Table 3.** Top 10 common reasons for consultation with primary health care in the year before suicide date for the case group or the index date for the control date

**Rank**	**Case group (*n* = 14 515)**	**Control group (*n* = 580 159)**
**By period prevalence, *n* (%)^a^**		
1	Medication review and requests 1720 (11.85)	Medication review and requests 49 915 (8.60)
2	Depression 1065 (7.34)	Pain complaints 33 968 (5.85)
3	Pain complaints 918 (6.32)	Cough 11 872 (2.05)
4	Mental health assessment and care 598 (4.12)	Hypertension 11 380 (1.96)
5	Overdose 446 (3.07)	Influenza vaccination 9940 (1.71)
6	Anxiety 408 (2.81)	Contraception 9713 (1.67)
7	Self-inflicted injury 390 (2.69)	Asthma 8928 (1.54)
8	Cough 268 (1.85)	Diabetes 8353 (1.44)
9	Sleep disturbance 260 (1.79)	Chest infection 7993 (1.37)
10	Asthma 235 (1.62)	Depression 6321 (1.09)

**By total records, *n* (per 100 person-years)^b^**		
1	Medication review and requests 4890 (33.69)	Medication review and requests 107 082 (18.46)
2	Depression 2610 (17.98)	Pain complaints 56 627 (9.76)
3	Pain complaints 1707 (11.76)	Diabetes 20 124 (3.47)
4	Mental health assessment and care 1355 (9.34)	Hypertension 19 415 (3.35)
5	Anxiety 859 (5.92)	Cough 16 434 (2.83)
6	Wound 851 (5.86)	Wound 15 727 (2.71)
7	Overdose 655 (4.51)	Contraception 15 591 (2.69)
8	Self-inflicted injury 443 (3.05)	Asthma 13 440 (2.32)
9	Diabetes 419 (2.89)	Depression 12 061 (2.08)
10	Cough 412 (2.84)	Influenza vaccination 10 410 (1.79)

a *Period prevalence of reason for consultation, that is, the proportion of case patients and control patients with at least one record of a consultation for that reason in the final year.*

b *Total recorded consultations for that reason in the final year.*

For the control group, the most common reasons for consultation in the final year were medication reviews and requests and pain complaints (by period prevalence and total records) followed by cough (by period prevalence) and diabetes (by total records). Patients who died by suicide were more likely to consult in the final year for mental health issues (including anxiety), self-harm (including overdose), and sleep disturbances.

## Discussion

### Summary

This large population-based, nationally representative longitudinal study has shown that, for patients who died by suicide, primary care consultation rates gradually increased throughout the preceding 5 years, particularly in the 3 months before suicide, whereas consultation rates remained static for control patients. The risk of suicide was six times higher in patients who consulted more than once per month in the final year, compared with those consulting only once per year. A higher risk in those consulting more than once per month (ranging from three- to ninefold) was shown across all sociodemographic groups as well as in patients with, and to a lesser degree, without psychiatric illnesses (60% increase), when compared with equivalent groups consulting once annually. The most common reason for consulting before suicide for both the case and the control groups was a medication review.

### Strengths and limitations

There are several strengths in this study. The large size of a nationally representative sample and the high specificity of suicide death records obtained from national mortality registries were major strengths. Additionally, the longitudinal collection of patient records without prior knowledge of the hypothesis of interest means that bias was potentially minimal. For the first time, to the authors’ knowledge, the vast and intricate clinical information deposited in CPRD were systematically approached to derive clinically meaningful categories denoting reasons for consultations.

However, there are limitations to this study. In this study the authors used consultation patterns with primary care to identify patients at higher risk of suicide, but it was not possible to analyse other important factors that may differentially influence those patterns, such as access to primary health care. Moreover, medical terms in CPRD do not distinguish between a diagnosis and a patient-reported symptom. Although this is a potential limitation in the study, examining coded medical terms does reflect the real-world data available to primary care clinicians when reviewing notes during a consultation and as such ensures the applicability of the findings to everyday consultations. Future work exploring consultations by presenting complaint, rather than recorded diagnosis, as well as further exploring consultation patterns by different modes of consultation (for example, telephone or face-to-face) and with different healthcare professionals is warranted.

### Comparison with existing literature

In line with previous reports,^1^^–^^4^^,^^6^^–^^8^^,^^21^^,^^22^ the majority of individuals who died from suicide in the current study consulted with primary care professionals at least once in their final year. The results also concur with earlier studies, suggesting high suicide risk is associated with frequent consultations with primary care.^5^^–^^7^ However, the current study has extended knowledge of this topic by investigating suicide risk in relation to consultation patterns in different sociodemographic and clinical groups.

Although females, younger individuals, and those experiencing less socioeconomic deprivation are generally known to be at a relatively lower risk of suicide,^23^^–^^25^ the current study found that those groups were the ones in which increased suicide risk was most identifiable via high primary care use. In part, this may relate to higher primary care consultation rates among such groups. Females have higher consultation rates than males, most marked in the 21–39 years age group, whereas males consult 60% less.^26^ Certain conditions known to be more prevalent in younger and female populations, most importantly self-harm,^27^^–^^29^ may also contribute to more consultations and could explain this prominence in suicide risk demonstrated. It is possible that those from less deprived backgrounds with recurrent medical problems will be more likely to consult than their counterparts from more deprived backgrounds.

Unlike the results from Windfuhr and colleagues, showing an increased suicide risk in those who did not consult in the year before suicide,^7^ the current study showed a significantly reduced risk in this group. The type of consultation included in the study may have modified this risk, as Windfuhr and colleagues only examined face-to-face consultations, whereas the current study included other types of live consultation. It may be that patients not involved in face-to-face clinical encounters represent a subgroup of patients with multiple disadvantages such as homelessness, offending, substance misuse, chronic poverty, and/or mental health problems.^30^ This could make them more liable to suicide risk, possibly owing to the interplay between disengagement with healthcare services and factors directly related to the multiple disadvantages.

Similar to findings from previous research,^4^^,^^8^ this study observed that, in the year before suicide, consultation rates by patients who died by suicide were not only higher than those of the control patients, but were steadily increasing over time, particularly in the final 3 months. This study has additionally illustrated that a similar pattern exists throughout the 5-year period before death by suicide. This means that there is potential space for effective preventive measures years before suicide occurs. The authors speculate that frequent consultations could indicate increases in disease severity, unresolved medical complaints, or ongoing psychosocial stressors manifesting as psychological or physical complaints.

The common reasons why patients who died by suicide consulted before their death included mostly modifiable factors (such as depression and pain). Apart from self-inflicted injury and overdose, the other top 10 reasons for consultation by patients who died by suicide were not explicitly linked to suicide but were temporally associated with the event. Most of these, such as depression and anxiety,^31^ sleep disturbance,^32^ and pain^33^^,^^34^ have previously been shown to be associated with higher suicide risk. The authors of the current study did not have data on whether suicidal thoughts were discussed in these consultations or not, but perceived barriers such as stigma^35^^,^^36^ and unwanted consequences such as involuntary admissions^36^^,^^37^ could have played a role in hindering disclosure of suicidal thoughts and behaviours.

### Implications for practice

On recognising increases in consultation frequency, particularly to more than once per month, the authors recommend that primary healthcare clinicians consider the possibility of psychiatric illnesses and suicide risk, alongside usual attempts to optimise management of existing conditions. Based on the results in the current study, and other existing evidence,^31^^,^^33^^,^^34^^,^^38^ it is important that consultations for depression, anxiety and other mental health conditions, sleep disturbance, and pain complaints, especially in the context of increasing consultation frequency, include screening for suicidal thoughts, with a more thorough risk assessment if these are exhibited.

A medication review is indeed the most common reason for consulting and is primarily a routine appointment often requested by the clinical team rather than the patient. Thus, clinicians should use routine monitoring appointments, such as medication reviews, to consider psychiatric illnesses, especially depression, as evidence has shown that improved recognition and treatment of depression by healthcare providers can help reduce suicide.^39^^,^^40^

In conclusion, an increase in consultation rates with primary care is recognised in individuals who died from suicide during the preceding 5 years, climaxing in the final 3 months. Consulting more than once per month was associated with a particularly increased risk. As well as patient-initiated consultations, routine monitoring appointments, such as medication reviews, should be viewed as a key opportunity to assess for psychiatric conditions known to increase suicide risk.

## Data

The data used for this work were obtained under licence from Clinical Practice Research Datalink (CPRD). This licence does not permit further sharing. However, anyone wishing to access the data can obtain it directly from CPRD subject to their licensing requirements.
